# Release of Matrix Metalloproteinases-2 and 9 by S-Nitrosylated Caveolin-1 Contributes to Degradation of Extracellular Matrix in tPA-Treated Hypoxic Endothelial Cells

**DOI:** 10.1371/journal.pone.0149269

**Published:** 2016-02-16

**Authors:** Haoming Song, Youjun Cheng, Gang Bi, Yihui Zhu, Wei Jun, Wenlin Ma, Huimin Wu

**Affiliations:** 1 Department of Cardiology, Tongji Hospital, Tongji University School of Medicine, Shanghai 200065, China; 2 Department of Neurology, Guangzhou Brain Hospital, Affiliated Hospital of Guangzhou Medical University, Guangzhou 510370, China; 3 Translational Center for Stem Cell Research, Tongji Hospital, Stem Cell Research Center, Tongji University School of Medicine, Shanghai 200065, China; 4 Department of orthopedics, Tongji Hospital, Tongji University School of Medicine, Shanghai 200065, China; 5 Department of general surgery, Tongji Hospital, Tongji University School of Medicine, Shanghai 200065, China; National Institutes of Health, UNITED STATES

## Abstract

Intracranial hemorrhage remains the most feared complication in tissue plasminogen activator (tPA) thrombolysis for ischemic stroke. However, the underlying molecular mechanisms are still poorly elucidated. In this study, we reported an important role of caveolin-1 (Cav-1) s-nitrosylation in matrix metalloproteinase (MMP)-2 and 9 secretion from tPA-treated ischemic endothelial cells. Brain vascular endothelial cells (bEND3) were exposed to oxygen-glucose deprivation (OGD) for 2 h before adding recombinant human tPA for 6 h. This treatment induced a significant increase of MMP2 and 9 in the media of bEND3 cells and a simultaneous degradation of fibronectin and laminin β-1, the two main components of extracellular matrix (ECM). Inhibition of MMP2 and 9 with SB-3CT completely blocked the degradation of fibronectin and laminin β-1. ODG+tPA treatment led to Cav-1 shedding from bEND3 cells into the media. Notably, OGD triggered nitric oxide (NO) production and S-nitrosylationof Cav-1 (SNCav-1). Meanwhile tPA induced activation of ERK signal pathway and stimulates the secretion of SNCav-1. Pretreatment of bEND3 cells with C-PTIO (a NO scavenger) or U0126 (a specific ERK inhibitor) significantly reduced OGD-induced S-nitrosylation of Cav-1 in cells and blocked the secretion of Cav-1 and MMP2 and 9 into the media as well as the degradation of fibronectin and laminin β-1 in OGD and tPA-treated cells. These data indicate that OGD-triggered Cav-1 S-nitrosylation interacts with tPA-induced ERK activation to augment MMP2 and 9 secretion and subsequent ECM degradation, which may account for the exacerbation of ischemic blood brain barrier damage following tPA thrombolysis for ischemic stroke.

## Introduction

Tissue plasminogen activator (tPA) is a serine protease that catalyzes the conversion of plasminogen to plasmin to dissolve fibrin-based blood clots[1]. Recombinant human tPA was approved by FDA to treat acute ischemic stroke in 1996 and remains to be the only stroke therapy almost two decades later. TPA thrombolysis is limited to less than 5% of stroke patients due to the narrow therapeutic time window (4.5 hrs after stroke onset) and the potentially devastating complication of intracerebral hemorrhage (ICH)[2–4]. TPA-associated ICH occurs as a consequence of severe blood brain barrier (BBB) disruption during thrombolytic reperfusion[5]. Several mechanisms have been proposed for the exacerbation of BBB injury following tPA administration, such as augmenting proteolytic degradation of BBB structural components,[6, 7], toxicity to neurovascular cells[8, 9], and increased free radical generation[10]. However, the molecular events of tPA application after ischemic stroke remain unknown.

There are two essential elements, extracellular matrix (ECM) and tight junction, to keep BBB integrity. Tight junction proteins mainly including occludin and claudins form the initial barrier at the endothelial cells between blood and brain cells[11]. ECM includes interstitial matrix and basement membrane which provides critical support for vascular endothelium and a scaffold essential for maintaining the organization of vascular endothelial cells into blood vessels[12]. Some major components of ECM such as Type-IV collagen, fibronectin, heparan sulfate, and laminin form the basal lamina to surround the abluminal surface of the endothelial cell[11]. The gelatinases matrix metalloproteinase-2 and 9 (MMP2 and 9) are synthesized inside the cells and secreted into extracellular space to digest occludin[13, 14], collagens, laminins, and fibronectin[15–18]. Increasing evidence shows that tPA is a stimulator and activator of MMP2 and 9 [1, 19, 20]. However, the knowledge about the interaction between tPA and MMP2 and 9 after ischemic stroke remains quite limit.

As a principal component of caveolae membranes in vivo, Caveolin-1 (Cav-1) has been improved to be involved in proteins transport[13, 21] and secretion[22–24]. Cav-1 contributes to BBB injury through interrupting tight junction proteins under several pathological conditions. Moreover, Cav-1 is secreted in a biologically active form by prostate cancer cells[25–27]. Although the expression of MMP2 and 9 has been regulated by Cav-1[28], it is unknown whether their secretion is regulated by Cav-1. And the mechanism of endothelial Cav-1 secretion is also unclear.

In this study, we investigated the effects of tPA treatment on MMP2 and 9 secretion in ischemic brain microvacular endothelial cells. Our results demonstrated that the addition of tPA to oxygen glucose deprivation (OGD)-treated endothelial cells promoted MMP2 and 9 secretion and Cav-1 shedding, which resulted in increased degradation of fibronectin and laminin β-1. Importantly, OGD-triggered nitric oxide (NO) production and Cav-1 S-nitrosylation and tPA-induced ERK activation critically contribute to the above changes.

## Materials and Methods

### Ethic Statements

The study was approved by the committees on ethical practice in Tongji University Hospital and the Health Office of China. All animal experiments were performed in accordance with the “Guidelines for Experimental Animals” at the Ministry of Science and Technology (Beijing, China). All dissections were performed according to recommendations proposed by the European Commission, and all efforts were made to minimize suffering in our animals.

### Cell culture and OGD treatment

Mouse brain microvascular endothelial cells (bEND3) (American Type Culture Collection) were grown as a monolayer in DMEM containing 15% fetal bovine serum, 100 U/ml penicillin, and 100 μg/ml streptomycin in a humidified incubator at 37°C with 5% CO_2_ and 95% room air. The cells were subcultured into 60 mm dishes coated with type I collagen and were grown to confluence before exposing to OGD for 2 h. Cell OGD treatment were performed as previously described[13]. Confluent bEND3 cells were transferred to glucose free medium (DMEM without glucose) pre-equilibrated with 95% N_2_ and 5% CO_2_, then incubated in a humidified airtight chamber (Billups-Rothberg Inc.) equipped with an air lock and flushed with 95% N_2_ and 5% CO_2_ for 15 min, and kept at 37°C for another 105 min. Control cultures were incubated with normal DMEM medium without FBS for 2 h at 37°C in 95% air and 5%. After OGD treatment, the cells were incubated with 20 μg/ml recombinant human tPA (Genentech) for 6 h before collecting the media and cellular extracts for further analyses. For mechanistic studies, MMP2 and 9 specific inhibitor SB-3CT (10 μM, Calbiochem) or specific ERK phosphorylation inhibitor U0126 (0.5 U/ml, Sigma) was applied at the beginning of tPA treatment, while Carboxy-PTIO (C-PTIO) (10 μM, Cayman), a scavenger of NO, was added to cells at the same times of OGD treatment.

### Isolation of insoluble fractions from conditioned media

To assess the levels of Cav-1 and MMP2 and 9 in the conditioned media after indicated treatment, insoluble fractions were isolated from the conditioned media according to the method described by Inoue et al.[29]. In brief, the media yielded S1 supernatant and P1 pellets after two 10-min centrifugations at 800 × g. S2 supernatant and P2 pellet were obtained after S1 supernatant was centrifuged at 10000 × g for 20 min. The S2 supernatant was further centrifuged at 100000 × g for 3 h and yielded S3 supernatant and P3 pellet. Cav-1 and MMP2 and 9 in P3 pellet were detected by Western blot and gel gelatin zymography, respectively.

### Knockdown of Cav-1 with siRNA

The bEND3 cells at 60%–70% confluence were transfected using 80 pmol of Cav-1 siRNA (Santa Cruz Biotech.) or scrambled control siRNA (Santa Cruz Biotech.) using siRNA transfection reagent (Santa Cruz Biotech.) following the manufacturer’s instruction. Forty-eight hours after transfection, the cells were subjected to OGD, tPA, or OGD+tPA treatment. Specific silencing was confirmed by Western blot.

### Preparation of total cell lysates and Western blot

After treatment, the cells were washed three times with pre-cooled PBS, and the total protein of cell lysates was extracted with RIPA buffer (Santa Cruz Biotech.). The specific protein was detected by Western blot analysis as previously described[13]. In brief, the cell lysates or S3 pellet in media were boiled and then electrophoresed in 12% SDS-PAGE acrylamide gels. They were then transferred onto nitrocellulose membranes (Bio-Rad) and incubated for 1 h in TBS-T containing 5% nonfat milk. The membranes were incubated overnight at 4°C with primary antibodies against fibronectin (Santa Cruz Biotech.), laminin β-1 (Santa Cruz Biotech.), Cav-1 (Santa Cruz Biotech.), and ERK phosphorylation (Cell Signaling). The membranes were washed three times in TBS-T and incubated for 1 h at room temperature with corresponding HRP-conjugated anti-rabbit or anti-mouse antibodies (Santa Cruz Biotech.). The membranes were developed with the SuperSignal West Pico HRP substrate kit (Pierce) and photographed on a Kodak 4000 image station (Carestream Molecular Imaging). To control sample loading and protein transfer, we stripped and reprobed the membranes with β-actin (Santa Cruz Biotech.) or total ERK (Cell Signaling) antibody.

### Gel gelatin zymography

After OGD treatment, MMP2 and 9 in cellular extracts, conditioned media, and insoluble fractions were analyzed by gelatin zymography as previous description[13, 30]. Equal amounts of proteins were concentrated with gelatin-sephrose 4B beads (GE Healthcare). The eluted MMP2 and 9 were electrophoretically separated on 10% SDS-polyacrylamide gels co-polymerized with 1 mg/ml gelatin (Sigma) under nonreducing conditions. The gels were washed in 2.5% Triton X-100 and then incubated for 48 h with a developing buffer containing 50 mM Tris (pH 7.6), 5 mM CaCl_2_, 0.2 mM NaCl, and 0.02% (w/v) Brij-35 at 37°C. Gels were stained with 0.125% Coomassie blue R-250 for 30 min and destained to visualize gelatinolytic bands (MMP2and 9) on a dark blue background. The intensities of MMP2 and 9 bands were analyzed using the Kodak 4000 image station (Carestream Molecular Imaging). A mixture of human MMP2 and 9 (Chemicon) was used as gelatinase standards.

### Real-time RT-PCR

Total cellular RNA was isolated, and real-time RT-PCR was performed according to our previous method[13]. The primers for MMP-2, MMP-9, and GAPDH were the same as previously reported[13]. The primers for Cav-1 (NM_031556) were as follows: forward: 5′-GTGAATGAGAAGCAAGTGTACG-3′ and reverse: 5′-AGATGCCGTCGAAACTGTG-3′. All primers were designed and synthesized by Integrated DNA Technologies. The fluorescence threshold value (Ct value) was calculated using SDS Enterprise Database software (Applied Biosystems). The relative value of mRNA expression was calculated by the comparative ΔΔC_t_ method as previous description[30]. In brief, mean C_t_ values were normalized to GAPDH (internal control), and the difference was defined as ΔC_t_. The difference between the mean ΔC_t_ values of treated and untreated cells was calculated and defined as ΔΔC_t_. The comparative mRNA expression level was expressed as 2^−ΔΔCt^.

### Immunostaining of Cav-1 in bEND3 cells

The bEND3 cells grown to confluence on collagen-coated coverslips were subjected to the indicated treatments. The cells were washed three times with PBS, fixed in 4% paraformaldehyde for 10 min, permeabilized with 0.1% Triton X-100 for 5 min, and then blocked for 1 h at room temperature with 3% BSA + 0.1% Tween-20 + 5% goat serum. The cells were incubated with anti-Cav-1 primary antibodies (1:100 dilution, Santa Cruz Biotech.) at 4°C overnight and then incubated with Cy3-conjugated anti-rabbit second antibody (1:200 dilution, Invitrogen) for 60 min. Coverslips were washed with PBS and mounted on glass slides with anti-fade solution Vectashield (Vector Laboratories). Images were acquired using an LSM 510 confocal laser-scanning microscope (Zeiss).

### Measurement of NO in endothelial cells

The NO-specific fluorescent dye 4,5-diaminofluorescein diacetate (DAF-2DA, Calbiochem) was used to detect NO production by bEND3[31]. After each indicated treatment, DAF-2DA (5 μM) was added to the media of bEND3 cells for 20 min at 37°C. The cells were rinsed three times, stored in the dark, and maintained at 37°C with a warming stage (Bioptechs, Inc.) on a Zeiss Axiort S100 TV inverted microscope (Carl Zeiss Inc., Thornwood, NY). Green fluorescence intensity was quantified using IP Labs software (Scanalytics Inc., Fairfax, VA). Data for each experiment were normalized to a reference image of the basal state.

### Measurement of protein S-nitrosylation

Biotin-switch method was used to detect S-nitrosylation of Cav-1 according to the protocol of *S-*nitrosylation protein detection assay kit (Cayman) and to a previously described method[32]. The cells were harvested in 1 ml wash buffer after washing four times with cold PBS. Protein-matched aliquots of cell lysates were labeled with biotin after blocking free thiols and reducing *S*-NO bonds. The final yield of the biotinylated proteins was dissolved in 100 μl of wash buffer and separated from nonbiotinylated proteins using NeutrAvidin agarose resin (Thermo Scientific, Rockford, IL) packed in centrifuge minicolumns. Equal volumes of each elute fraction were used for Western blot analysis after the bound proteins were eluted with a buffer. Input of the prebiotinylation cell lysates was also detected by Western blot.

### Statistical analysis

All data were presented as means ± SE. ANOVA followed by Tukey’s *post hoc* test was carried out to assess the differences between groups. The difference between two groups was statistically analyzed by Student *t* test. *p* ≤ 0.05 was considered statistically significant.

## Results

### Administration of tPA after 2 h OGD reduces fibronectin and laminin β-1 and increases MMP2 and 9 in the media of endothelial cells

**Fig 1:**
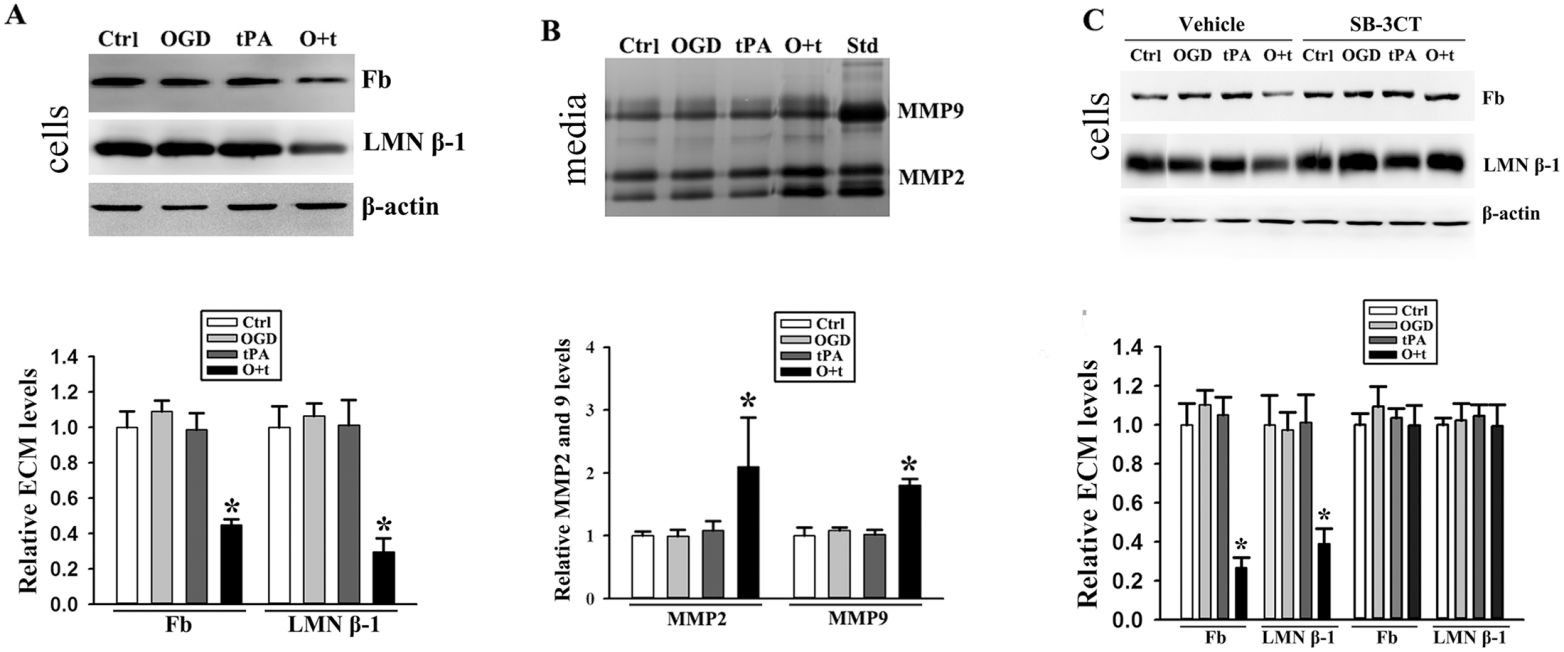
tPA promotes MMP2 and 9-mediated fibronectin and laminin β-1 degradation in OGD-treated endothelial cells. ***A)*** The levels of fibronectin (Fb) and laminin β-1 (LMN β-1) significantly decreased in bEND3 that were first exposed to 2-h OGD and followed by tPA treatment for 6 h (O+t) compared with untreated cells (Ctrl) or the cells that were treated by OGD or tPA alone. Upper panel: representative immunoblots, β-actin served as loading control; Bottom panel: band intensity was quantitated after normalization to β-actin and the data were expressed as mean ± SD, *p < 0.05, ANOVA, N = 4. ***B)*** Gelatin zymography analysis showed that OGD+tPA treatment (O+t) significantly increased MMP2 and 9 levels in the conditioned media. Upper panel: representative gelatin zymograms; Bottom panel: band intensity was quantitated and the data were expressed as mean ± SD, *p < 0.05, ANOVA, N = 4. Std: human MMP2 and 9 standards. ***C)*** SB-3CT completely blocked OGD+tPA (O+t)-induced degradation of fibronectin (Fb) and laminin β-1 (LMN β-1). Left panel: representative immunoblots; Right panel: band intensity was quantitated after normalization to β-actin and the data were expressed as mean ± SD, ANOVA, N = 4.

To mimic the thrombolytic treatment for acute ischemic stroke, recombinant human tPA (20 μg/ml) was added to bEND3 cells that were pretreated with OGD for 2 h. To exclude that part of the proteins found in the culture medium are due to cell death, we detected the cell toxicity using a CytoTox 96 Nonradioactive Cytotoxicity Assay Kit (Promega) and no cytotoxicity was found after tPA treatment (data not shown)[13]. After tPA treatment for 6 h, fibronectin and laminin β-1 were assessed by Western blot. As shown in Fig 1A, both fibronectin and laminin β-1 were significantly decreased in OGD+tPA-treated cells. tPA-induced upregulation of MMP2 and 9 has been reported in human cerebral microvascular endothelial cells[33]. Thus, we tested MMP2 and 9 levels in the media by gel gelatin zymography. Representative zymograms showed that the proteolytic bands representing pro- and active forms of MMP2 and 9 were detected under all tested experimental conditions (Fig 1B upper panel). Quantitative data showed that the addition of tPA to OGD-treated bEND3 cells induced significant increases of MMP2 and 9 in the media (Fig 1B, Lower panel). MMP3 level did not change (data not shown), although it has been previously reported to be involved in tPA-induced effects on endothelial cells[34]. To determine whether MMP2 and 9 are responsible for ECM degradation, we co-incubated the cells with tPA and the specific MMP2 and 9 inhibitor SB-3CT after 2-h OGD exposure. The results showed that SB-3CT completely blocked fibronectin and laminin β-1 degradation in tPA and OGD-treated cells (Fig 1C). These data indicated that tPA promoted MMP2 and 9 secretion in ischemic endothelial cells, resulting in increased ECM degradation(S1 Table).

### tPA-induced ERK Activation stimulates MMP2 and 9 expression and their secretion into conditioned media

**Fig 2:**
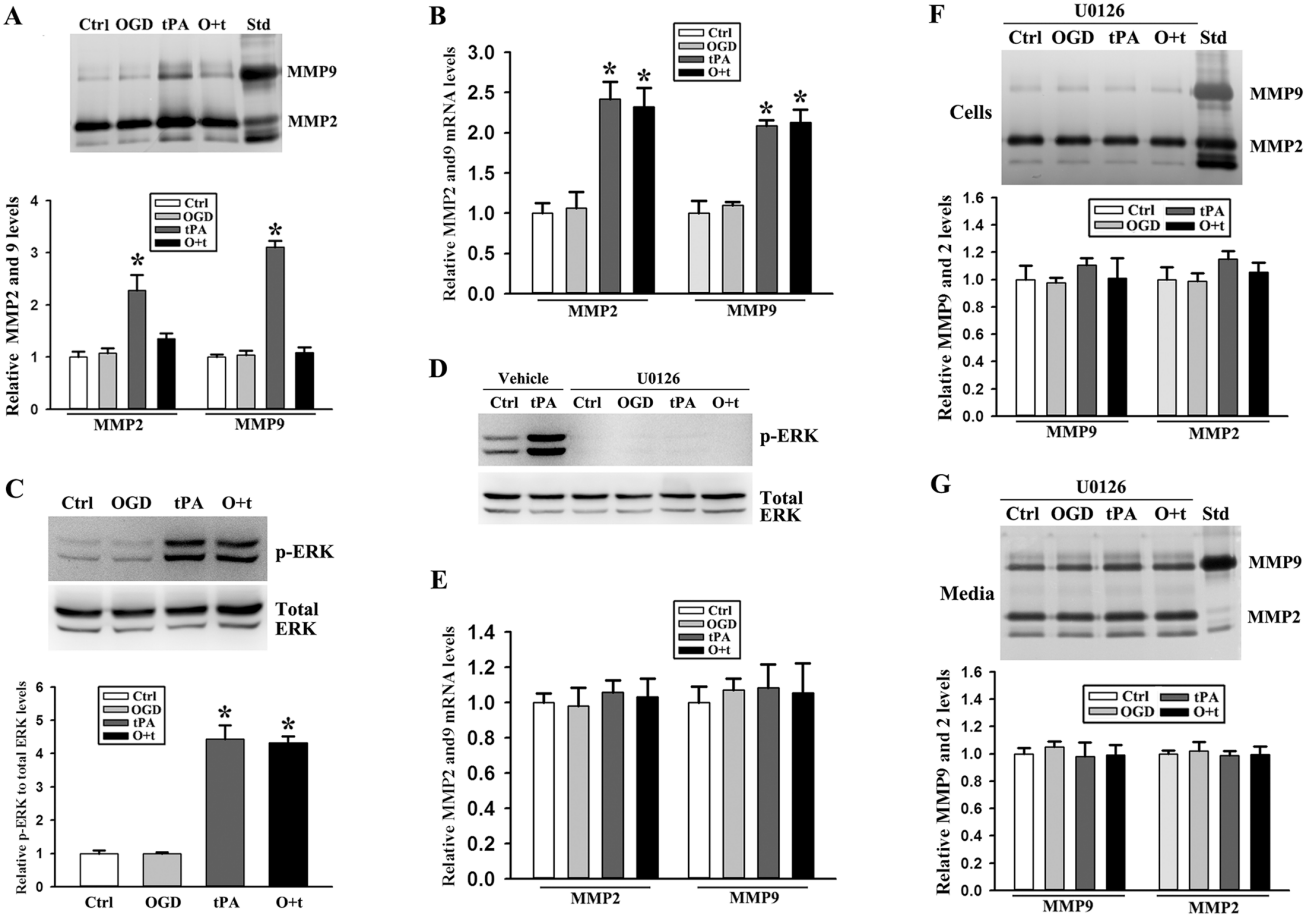
ERK activation mediates MMP2 and 9 upregulation and their release in tPA and OGD-treated endothelial cells. ***A)*** Gelatin zymography analysis showed that tPA alone significantly increased intracellular MMP2 and 9 levels, whereas their levels did not increase if tPA was administrated after OGD treatment (O+t). Upper panel: representative zymograms; Bottom panel: band intensity was quantitated and the data were expressed as mean ± SD, *p < 0.05, ANOVA, N = 3. Std: human MMP2 and 9 standards. ***B)*** Real time RT-PCR analysis showed that single tPA or tPA+OGD treatment (O+t) stimulated MMP2 and 9 mRNA expression in bEND3 cells. *p < 0.05, ANOVA, N = 4. ***C)*** tPA and OGD+tPA (O+t) treatment induced ERK phosphorylation (p-ERK). *p < 0.05, ANOVA, N = 5. ***D)*** U0126 completely blocked ERK activation. N = 3. ***E)*** TPA-induced MMP2 and 9 mRNA upregulation was inhibited by U0126. N = 3. ***F)*** The difference of MMP2 and 9 in cells among control (Ctrl), OGD, tPA, and OGD+tPA (O+t) was exterminated by U0126. Upper panel: representative zymograms; Bottom panel: band intensity was quantitated and the data were expressed as mean ± SD, ANOVA, N = 3. Std: human MMP2 and 9 standards. ***G)*** U0126 stopped OGD+tPA (O+t)-induced release of MMP2 and 9 in the media. Upper panel: representative zymograms; Bottom panel: band intensity was quantitated and the data were expressed as mean ± SD, ANOVA, N = 3. Std: human MMP2 and 9 standards.

MMP2and 9 are synthesized intracellularly and secreted into the media, therefore their increase in the conditioned media could be due to increased MMP-2/9 synthesis or increased secretion or both. To further clarify the mechanism underlying MMP2 and 9 increase in the conditioned media of tPA+OGD-treated endothelial cells, we assessed intracellular MMP2 and 9 levels and their mRNA expression. As shown in Fig 2A, intracellular MMP2 and 9 levels only increased in tPA-treated bEND3 cells. Interestingly, real-time RT-PCR showed that MMP2 and 9 mRNA expressions were increased in cells treated with tPA alone or OGD+tPA (Fig 2B). These data clearly indicated that tPA but not OGD stimulated MMP2 and 9 expression and synthesis in endothelial cells, while pre-exposure of endothelial cells to OGD promoted MMP2 and 9 secretion into the conditioned media.

We next investigated signal molecules that mediated tPA-induced MMP2 and 9 expression. ERK1/2 signal pathway can be activated by a diverse range of extracellular stimuli[35–37] and has been shown to mediated MMP2 and 9 expression[38, 39]. Therefore, we assessed ERK activation (phosphorylation) using Western blot and found that tPA alone or tPA + OGD induced ERK phosphorylation (Fig 2C). When ERK activation was inhibited by U0126 (Fig 2D), MMP2 and 9 mRNA upregulation was blocked in tPA-treated cells (Fig 2E), supporting an important role of ERK in tPA-induced MMP-2/9 expression.

Moreover, along with its inhibition on MMP-2/9 mRNA expression, the addition of U0126 also blocked tPA-induced intracellular MMP-2/9 increase (Fig 2F) as well as MMP-2/9 secretion in OGD + tPA-treated cells (Fig 2G). These results suggest that ERK signal pathway played an important role in mediating tPA-induced MMP-2/9 upregulation and tPA+OGD-induced MMP-2/9 secretion(S2 Table).

### Cav-1 contributes to MMP2 and 9 secretion in tPA+OGD-treated endothelial cells

**Fig 3:**
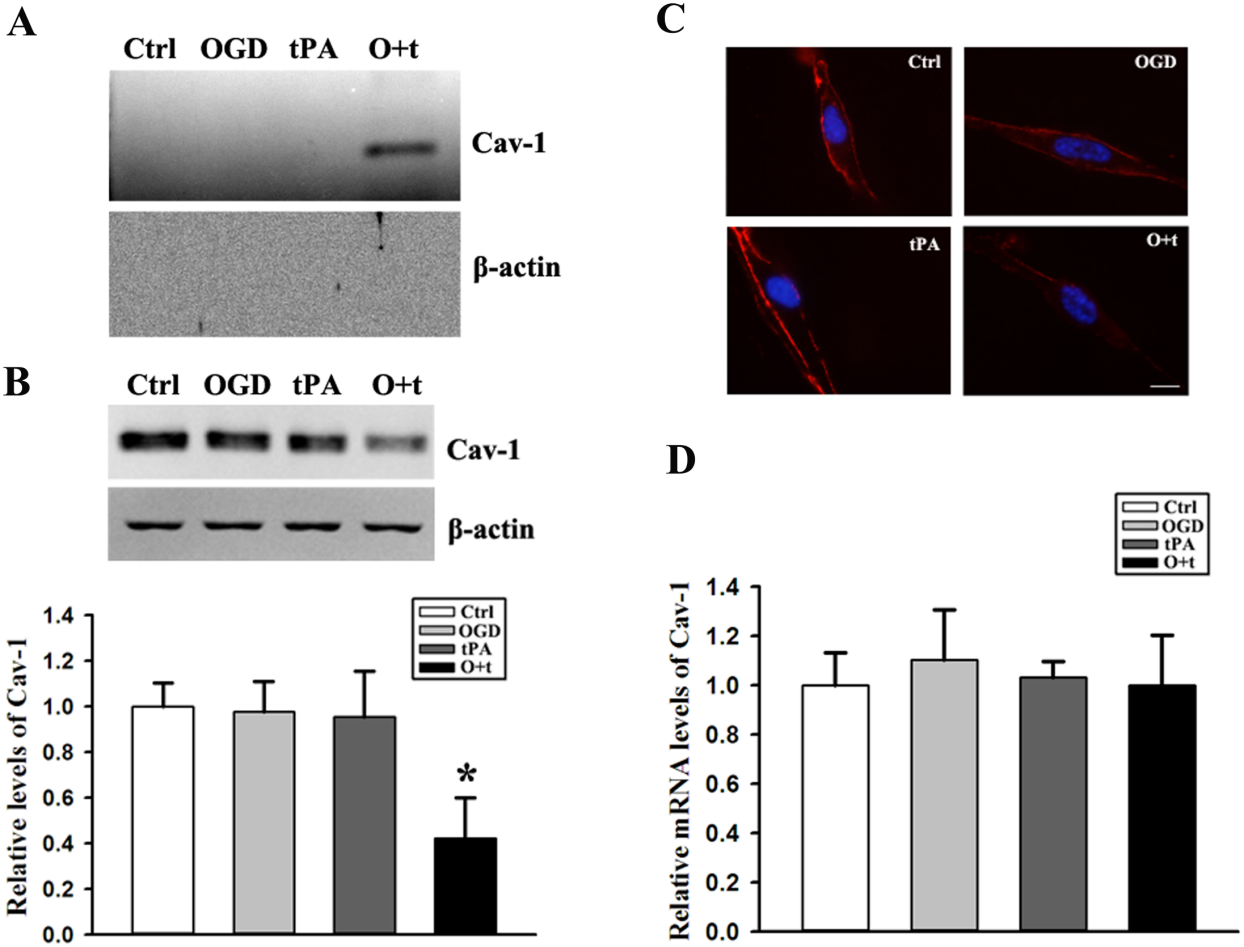
OGD+tPA treatment (O+t) promotes Cav-1 secretion from bEND cells. ***A)*** Upper panel: representative immunoblots showed that Cav-1 was detected in the conditioned media of tPA+OGD-treated cells (O+t), but not in untreated control cells (Ctrl) and the cells treated by OGD or tPA alone. Bottom panel: β-actin was not detected in the media of any cells. Experiments were repeated three times with similar results. ***B)*** Western blot analysis showed that OGD+tPA treatment (O+t) induced Cav-1 reduction in endothelial cells. Upper panel: representative immunoblots, β-actin served as loading control; Bottom panel: band intensity was quantitated after normalization to β-actin and the data were expressed as mean ± SD, *p < 0.05, ANOVA, N = 3. ***C)*** Confocal microscopic images revealed a circumcellular staining for Cav-1 protein in untreated control bEND3 cells, and OGD+tPA treatment (O+t) induced a significant reduction in Cav-1 immunostaining. Immunostaining experiments were repeated three times with similar results. Scale bar: 10 μm. ***D)*** Real time RT-PCR showed that OGD, tPA or their combination OGD+tPA (O+t) had no effect on Cav-1 mRNA expression level. Data were expressed as mean ± SD, ANOVA, N = 3.

To speculated that Cav-1 may mediate OGD+tPA-induced MMP2 and 9 secretion, Cav-1 in P3 pellets was measured by western blot, and the results showed that Cav-1 elevated in the media of tPA+OGD-treated cells, but not in untreated control cells and OGD or tPA-treated cells (Fig 3A, upper panel). The Cav-1 protein detected in the media was unlikely from detached endothelial cells because β-actin (a housekeeping protein) was not detected in any pellets isolated from the conditioned media (Fig 3A, bottom panel).

To further confirm whether increased Cav-1 in the media was from the cells, we assessed the changes of intracellular Cav-1 by western blot. As shown in Fig 3B, OGD or tPA alone did not change the total Cav-1 protein levels in bEND3 cells, however, their combination induced a significant reduction in the total protein level of Cav-1. To further verify these immunoblotting results, we performed immunofluorescence staining to visualize the change in Cav-1 under a confocal microscope. Confocal microscopic images revealed a circumcellular staining for Cav-1 protein in control bEND3 cells, regardless of whether they were pretreated with OGD or tPA. Consistent with the Western blot results, exposing cells to OGD+tPA significantly reduced the immunostaining of Cav-1 (Fig 3C). To rule out the possibility that OGD+tPA treatment could reduce Cav-1 synthesis, which in turn results in the reduction in total Cav-1 protein levels, we measured Cav-1 mRNA expression by real-time RT-PCR. OGD+tPA treatment did not affect Cav-1 mRNA expression (Fig 3D). These data indicated that tPA treatment could promote Cav-1 secretion in OGD-treated endothelial cells(S3 Table).

**Fig 4:**
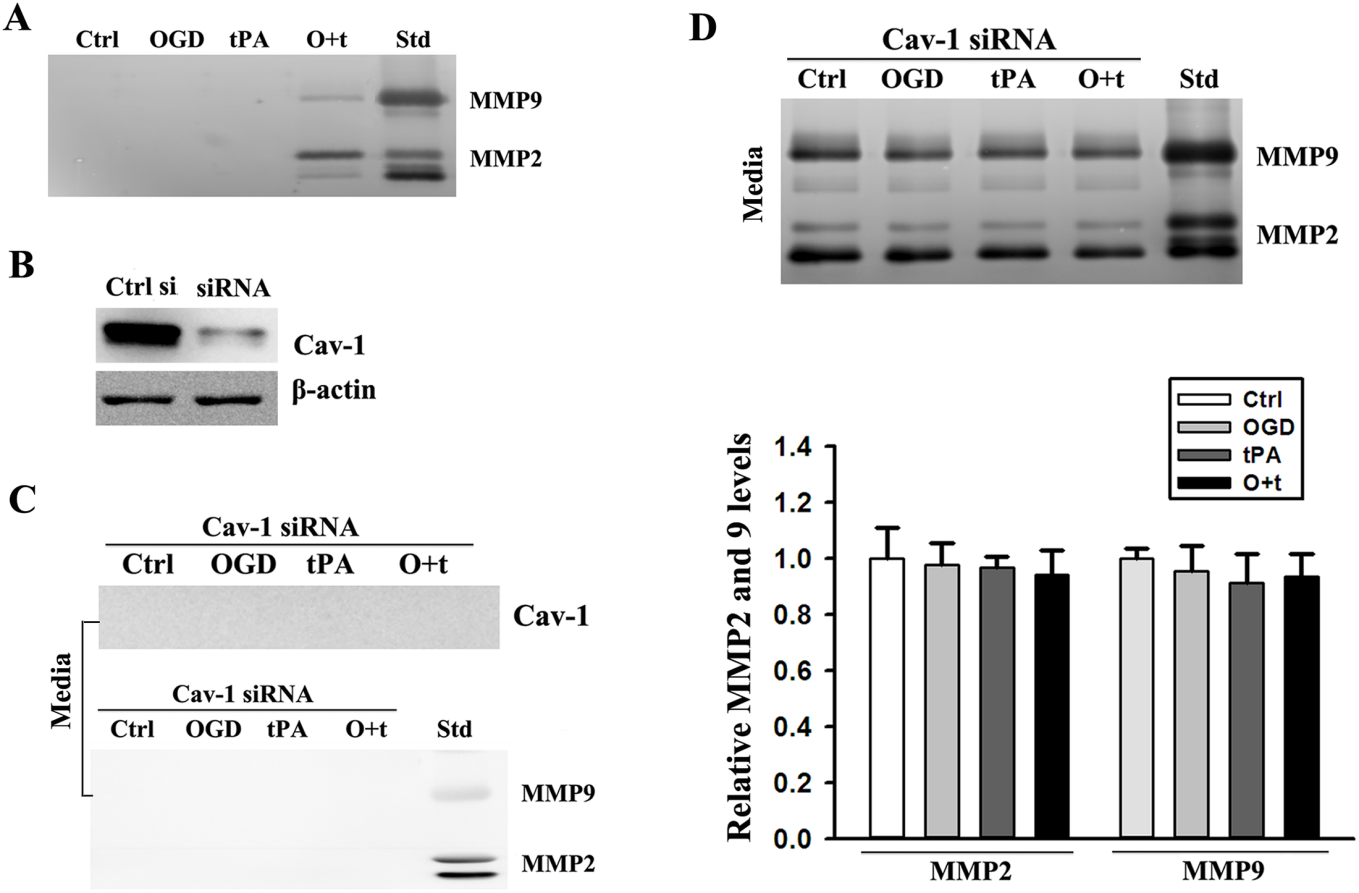
Caveolin-1 participated in the secretion of MMP2 and 9. ***A***) MMP2 and 9 existed in the insoluble fraction P3 of the media of cells treated with OGD+tPA (O+t). ***B***) Western blot analysis showed that Cav-1 siRNA effectively knocked down Cav-1 protein expression in bEND3 cells. Experiments were repeated three times and exhibited the same results. ***C***) Knockdown of Cav-1 with siRNA abrogated OGD+tPA (O+t)-induced existence of Cav-1 (upper panel) and MMP2 and 9 (bottom panel) in P3 in the media. Experiments were repeated three times and exhibited the same results. Std: human MMP2 and 9 standards. ***D***) Cav-1 siRNA recovered the same levels of MMP2 and 9 in the media of cells treated with OGD+tPA (O+t) and in the control (Ctrl), OGD, and tPA treatments. Upper panel: representative zymograms; Bottom panel: band intensity was quantitated and the data were expressed as mean ± SD, ANOVA, N = 3. Std: human MMP2 and 9 standards.

The levels of MMP2 and 9 in P3 pellet were examined by gel gelatin zymography. Similar to Cav-1 (Fig 3A), MMP2 and 9 were also found in the P3 pellet isolated from the conditioned media of with OGD+tPA-treated cells (Fig 4A). Moreover,The efficiency of Cav-1 siRNA in knocking down Cav-1 expression was assessed by western blot, and the result showed that Cav-1 siRNA resulted in a ~ 90% reduction in Cav-1 protein levels after 48 h of transfection (Fig 4B). Of note, when Cav-1 was knocked down, Cav-1 and MMP-2/9 were no longer detectable in the P3 pellets (Fig 4C), the increase of MMP2 and 9 in the conditioned media was also completely blocked OGD+tPA-treated cells (Fig 4D). It was worth pointing out that a significant amount of MMP-2/9 was still detected in the conditioned media of the cells exposed to the indicated treatments after Cav-1 siRNA transfection, suggesting that there were other mechanisms involved in MMP2 and 9secretion(S4 Table).

### OGD-induced NO mediates S-nitrosylation of Cav-1 and tPA-induced ERK activation stimulates the secretion of Cav-1

**Fig 5:**
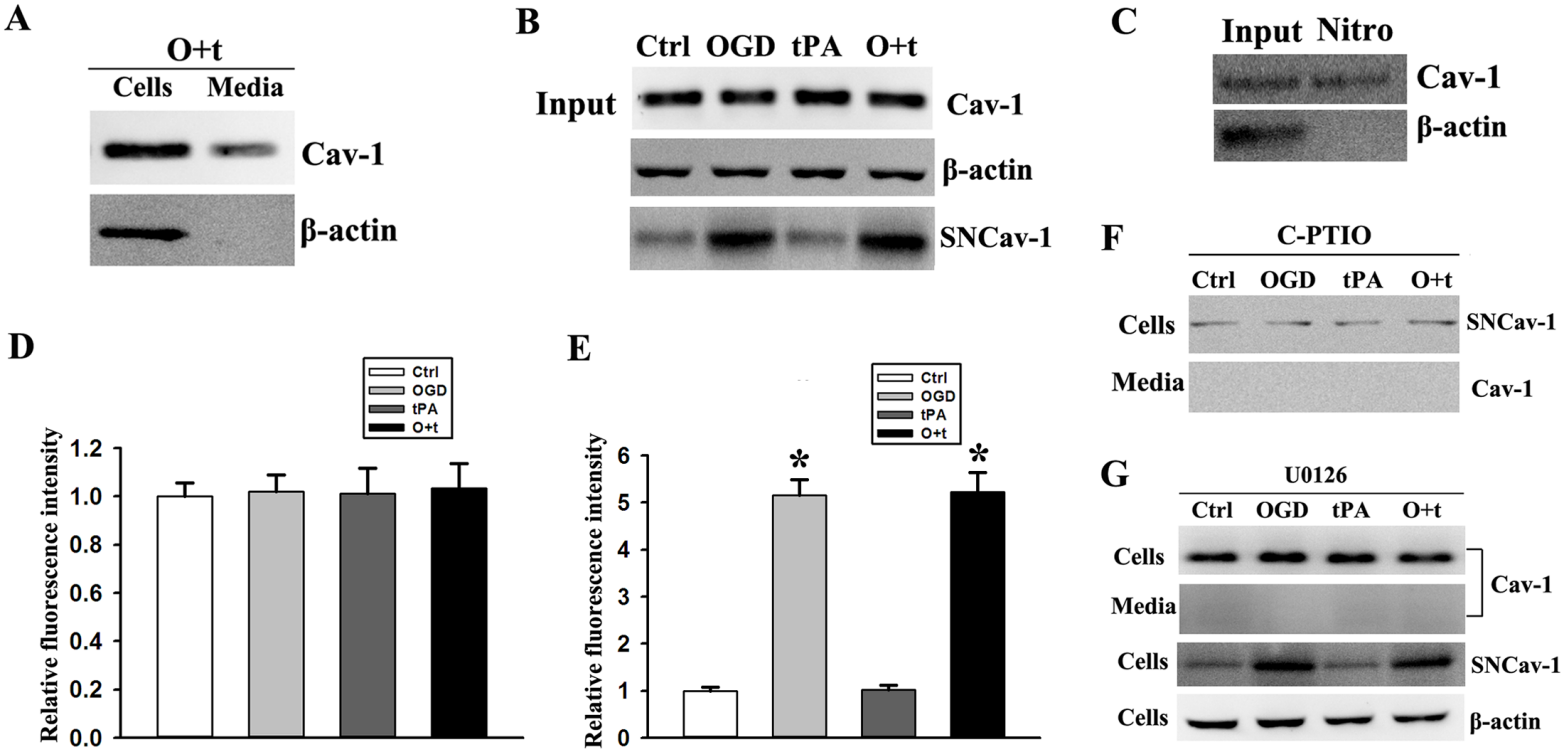
OGD-induced NO mediates S-nitrosylation of Cav-1 in bEND3 cells. ***A)*** Representative immunoblots revealed similar molecular weight of Cav-1 protein between cellular extracts and P3 pellets of the conditioned media following OGD and tPA treatment (O+t). ***B)*** S-nitrosylation of Cav-1 (SNCav-1) improved in the cells treated with OGD or OGD+tPA (O+t). Experiments were repeated three times and exhibited the same results. ***C)*** Cav-1 in the media of cells treated with OGD+tPA (O+t) was nitrosylated. Experiments were repeated three times and exhibited the same results. ***D)*** NO was measured in cells after 6 h of tPA treatment using a DAF-2DA kit. Low levels of NO and no significant difference were observed among the control (Ctrl), OGD, tPA, and OGD+tPA (O+t) treatment. The fluorescence intensity was quantitated after normalization to Ctrl and the data were expressed as mean ± SD, ANOVA, N = 5. ***E)*** NO was tested in cells after 2 h of OGD treatment, and NO increased in cells treated with OGD. The fluorescence intensity was quantitated after normalization to Ctrl and the data were expressed as mean ± SD, *p < 0.05 when compared with control (Ctrl), Student *t* test, N = 5. ***F)*** After NO was blocked with C-PTIO, S-nitrosylation of Cav-1 (SNCav-1) was inhibited in cells treated with OGD or OGD+tPA (O+t) (upper panel). Cav-1 in the media of cells treated with OGD+tPA (O+t) was not detected (bottom panel). Experiments were repeated three times and exhibited the same results. ***G)*** U0126 stopped the reduction of intracellular Cav-1 (upper first panel) and the secretion of Cav-1 from cells treated with OGD+tPA (O+t) (upper second panel). However, U0126 did not block S-nitrosylation of Cav-1 (SNCav-1) in cells treated with OGD or OGD+tPA (O+t) (upper third panel). Experiments were repeated three times and exhibited the same results.

TPA has been shown to cleave low-density lipoprotein receptor-related protein, resulting in its shedding[40]. we detected intracellular and extracellular Cav-1 of OGD+tPA-treated cells in the same SDS-PAGE gel. As shown in Fig 5A, intracellular and extracellular Cav-1 showed exactly the same molecular weights, suggesting that Cav-1 secretion did not relying on tPA cleavage. Although Cav-1 is secreted by some cancer cells and functions as a paracrine/autocrine factor, Cav-1 levels in endothelial-conditioned media were undetectable (Fig 3A). And to speculate that SNCav-1 may be an important trigger for Cav-1 secretion,we initially investigated the amino acid sequence of mouse Cav-1 and found that mouse Cav-1 had free cysteine (AA133, 143, 156), where S-nitrosylation occurred. We assessed the levels of SNCav-1 in the cellular extracts and conditioned media using the biotin-switch method. As shown in Fig 5B, SNCav-1 was increased in the cells that were treated with by OGD or OGD+tPA (Fig 5B). To determine whether the secreted Cav-1 was nitrosylated, we pooled P3 isolates from five dishes to obtain enough protein for biotin switch assay. The Western blot results showed that majority of Cav-1 in the conditioned media of OGD+tPA-treated cells were nitrosylated (Fig 5C).

To confirm that NO contributed to S-nitrosylation of Cav-1, we used DAF-2DA to assess NO production in bEND3 cells. When NO production was measured at 6 h after tPA addition, low levels of NO production were detected in all groups and there was no significant differences among untreated control cells, OGD-, tPA-, and OGD+tPA-treated cells (Fig 5D). Considering that NO is an atypical biomolecule with an ultra-short half-life and is highly diffusible when formed, we analyzed NO production immediately after OGD treatment and found that NO increased in the OGD-treated cells (Fig 5E). As positive control, incubation the cells with NONOate (10 μM, Cayman)led to a large amount of NO production (data not shown). To further determine whether Cav-1 S-nitrosylation observed above in OGD-treated cells was mediated by NO, we added NO scavenger C-PTIO to the media before OGD treatment. Indeed, the addition of C-PTIO abolished the increase of SNCav-1 levels in cells treated by OGD or OGD+tPA (Fig 5F, Upper panel). Of note, C-PTIO treatment led to the disappearance of Cav-1 in P3 pellets isolated from the media of OGD+tPA-treated cells (Fig 5F, Bottom panel).

Further more, to confirm that ERK might also mediate SNCav-1 secretion, we exposed bEND3 cells to OGD and tPA with the presence of ERK inhibitor U0126. As shown in Fig 5G (Upper panel), U0126 treatment completely blocked the reduction of intracellular Cav-1 in OGD+tPA-treated cells, which concurrently occurred with the disappearance of Cav-1 in the conditioned media (Fig 5G and 5A, upper second panel). Interestingly, U0126 failed to block Cav-1 S-nitrolysation in OGD-treated cells (Fig 5G, upper third panel). These results indicated that activation of ERK signal pathway was involved in SNCav-1 secretion, but not in SNCav-1 production in the cells exposed to OGD and tPA(S5 Table).

**Fig 6:**
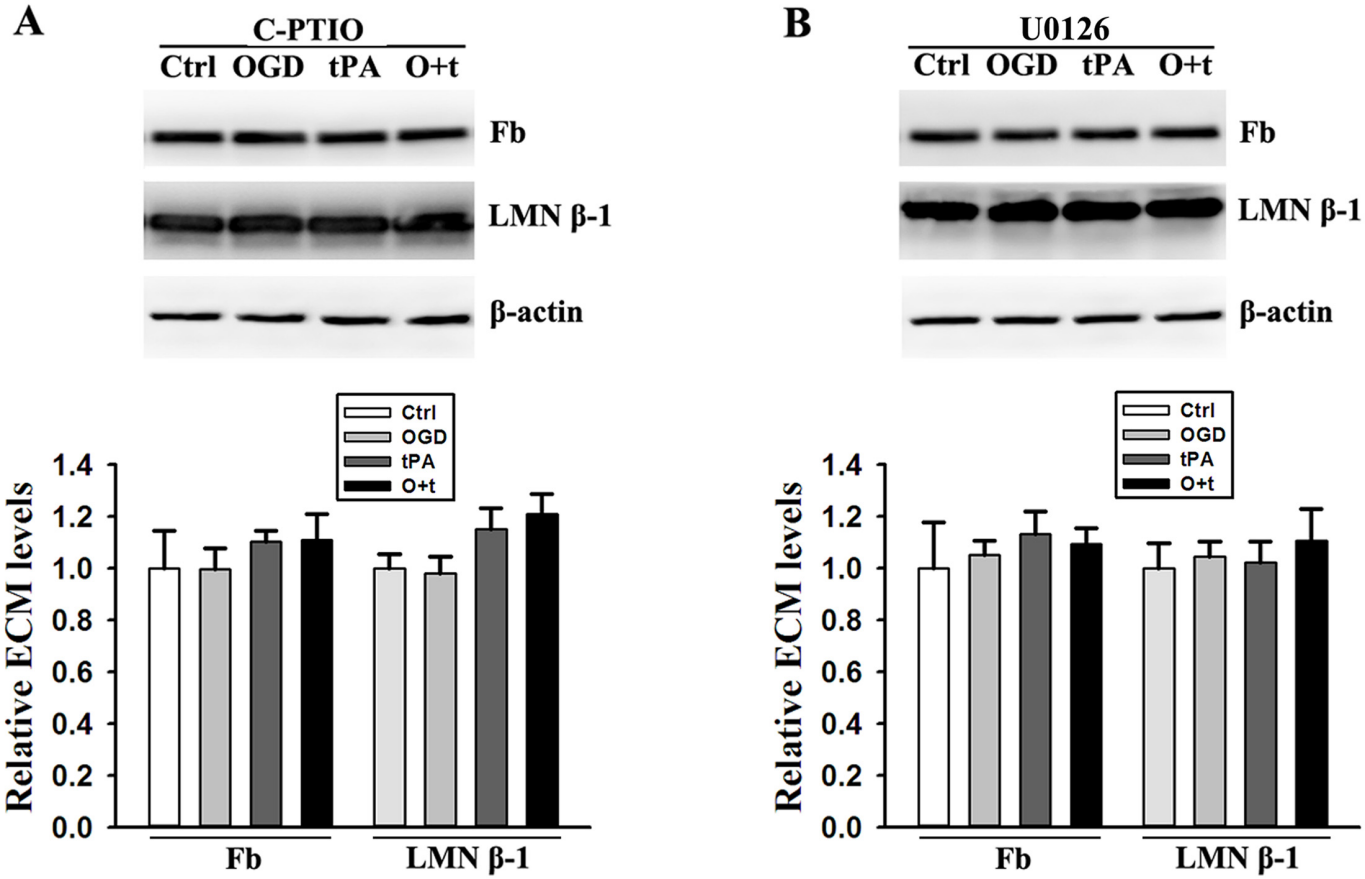
Scavenge of NO with C-PTIO (A) and inhibition of ERK signal pathway with U0126 (B) recover the same levels of fibronectin (Fb) and laminin β-1(LMN β-1) in the cells treated with control (Ctrl), OGD, tPA, and OGD+tPA (O+t). Upper panel: representative immunoblots, β-actin served as loading control; Bottom panel: band intensity was quantitated after normalization to β-actin and the data were expressed as mean ± SD, ANOVA, N = 4.

To further confirm that the release of MMP2 and 9 was induced by tPA administration after ischemia stroke and was associated with ECM degradation, we determined the fibronectin and laminin β-1 in cells treated with C-PTIO or U0126 using Western blot(S6 Table). OGD+tPA-induced degradation of fibronectin and laminin β-1 was blocked by NO scavenger with C-PTIO (Fig 6A) or inhibition of ERK phosphorylation with U0126 (Fig 6B).

## Discussion

ECM is a key structure in the subendothelial basement membrane of the vessel wall[41]. Therefore, breakdown of ECM as induced by MMP2 and 9 may be associated with intracerebral hemorrhage. In the present study, we observed that tPA alone induced expression of MMP2 and 9 in mouse bEND3, as revealed by increased mRNA and protein levels. However, applying tPA after OGD treatment (ischemic stroke *in vitro*) led to the enhancement of MMP2 and 9 mRNA levels, but not their protein levels. The tPA-induced MMP2 and 9 was released into the media when cells were pretreated with OGD. Of note, only OGD treatment did not induce secretion of MMP2 and 9, which is inconsistent with our previous findings[13]. The possible reason is that the induction of MMP2 and 9 secretion may terminate when OGD stops. Moreover, increase of MMP2 and 9 may disappear when the medium is displaced with fresh medium after 2h-OGD treatment. This is also refected by that NO can be detected only its production analyzed immediately after OGD treatment (Fig 5D and 5E). Meanwhile, fibronectin and laminin β-1, the two major components of ECM, were degraded in cells treated with OGD+tPA. Moreover, SB-3CT, a specific inhibitor of MMP2 and 9, completely blocked the degradation of fibronectin and laminin β-1. This finding confirms that tPA-induced release of MMP2 and 9 contributes to ECM degradation.

Increasing evidence suggests that Cav-1 plays a central role in maintaining the integrity of BBB through MMP regulation[42, 43]. We observed that Cav-1 appeared in the media of cells treated with tPA after OGD. Parts of MMP2 and 9 coexisted with Cav-1 in the insoluble fraction of the media. The increase of MMP2 and 9 in the media was eliminated after Cav-1 was knocked down by Cav-1 siRNA. These data suggest that Cav-1 directly participates in MMP2 and 9 trafficking.

We investigated how Cav-1 entered into the media of cells treated with tPA and OGD. Two crucial events occurred during OGD and continued tPA treatment. First, OGD-induced NO initiated S-nitrosylation of Cav-1. Second, tPA-induced activation of ERK signal pathway triggered the shedding of S-nitrosylated Cav-1. NO has an extremely short half-life and is an unstable gas[44, 45], but it is an important bioactive signaling molecule that is associated with protein nitrosylation. NO is directly implicated in BBB disruption and is an important messenger for intercellular and intracellular communication[46]. We found that Cav-1 in the media of cells treated with OGD+tPA occurred mainly in the form of S-nitrosylated Cav-1. These data suggest that NO-induced S-nitrosylation of Cav-1 during OGD is ready for shedding when cells continue to be stimulated by other factors, such as tPA.

Although most of the functions of tPA are driven by its serine-protease activity, growing evidence indicates that non-proteolytic activities are also important[47]. Some studies show that ERK signaling pathway can be activated by tPA[48, 49]. And the ERK/MAP kinase signaling cascade mediates ectodomain shedding of many transmembrane proteins[50, 51]. This study shows that tPA induced the activation of ERK signal pathway, as revealed by ERK phosphorylation. U0126 is a specific inhibitor for ERK phosphorylation that stopped the Cav-1 shedding of the cells treated with OGD+tPA into the media, resulting in no elevation of MMP2 and 9. Furthermore, C-PTIO, a NO scavenger, blocked both S-nitrosylation of Cav-1 and its appearance in the media of cells treated with OGD and tPA. However, U0126 only blocked Cav-1 shedding, but not the S-nitrosylation of Cav-1. These data strongly support that S-nitrosylation of Cav-1 is essential for Cav-1 shedding. Our finding shows for the first time that independently of its proteolytic activity, but by inducing phosphorylation of ERK, tPA can promote Cav-1 shedding.

Lastly, we determined that both U0126 and C-PTIO prevented fibronectin and laminin β-1 against degradation induced by OGD+tPA treatment. Combined with SB-3CT blocking OGD+tPA-induced degradation of fibronectin and laminin β-1, our results indicate that each step during OGD+tPA treatment is related to MMP2 and 9, which contributed to ECM degradation.

## Conclusion

In summary, our results demonstrate a novel mechanism underlying tPA-associated cerebral hemorrhage after ischemia. We propose that ischemia-induced NO initiates S-nitrosylation of Cav-1, and tPA-activated ERK signal pathway stimulates the expression of MMP2 and 9 and shedding of S-nitrosylated Cav-1. S-nitrosylation of Cav-1 switches the release of MMP2 and 9, which contributed to the degradation of fibronectin and laminin β-1. The findings in this study may provide clues for developing new treatment strategies to protect the ECM of vessels against degradation induced by MMP2 and 9 and thus safely expand tPA thrombolytic therapy for ischemia.

## Supporting Information

S1 Table The supporting data for Fig 1. TPA promotes MMP2 and 9-mediated fibronectin and laminin β-1 degradation in OGD-treated endothelial cells.
(XLSX)

S2 Table The supporting data for Fig 2. ERK activation mediates MMP2 and 9 upregulation and their release in tPA and OGD-treated endothelial cells.
(XLSX)

S3 Table The supporting data for Fig 3. Cav-1 contributes to MMP2 and 9 secretion in tPA+OGD-treated endothelial cells.
(XLSX)

S4 Table The supporting data for Fig 4. Caveolin-1 participated in the secretion of MMP2 and 9. ***A***) MMP2 and 9 existed in the insoluble fraction P3 of the media of cells treated with OGD+tPA (O+t).
(XLSX)

S5 Table The supporting data for Fig 5. OGD-induced NO mediates S-nitrosylation of Cav-1 in bEND3 cells.
(XLSX)

S6 Table The supporting data for Fig 6. The release of MMP2 and 9 induced by tPA administration after ischemia stroke.
(XLSX)
